# ROS-Induced GATA4 and GATA6 Downregulation Inhibits StAR Expression in LPS-Treated Porcine Granulosa-Lutein Cells

**DOI:** 10.1155/2019/5432792

**Published:** 2019-04-22

**Authors:** Xiaolu Qu, Leyan Yan, Rihong Guo, Hui Li, Zhendan Shi

**Affiliations:** ^1^Key laboratory of Animal Breeding and Reproduction, Institute of Animal Science, Jiangsu Academy of Agricultural Sciences, Nanjing 210014, China; ^2^Jiangsu Key Laboratory for Food Quality and Safety-State Key Laboratory Cultivation Base of Ministry of Science and Technology, Jiangsu Academy of Agricultural Sciences, Nanjing 210014, China; ^3^College of Animal Science and Technology, Jilin Agricultural University, Changchun 130118, China

## Abstract

LPS is a major endotoxin produced by gram-negative bacteria, and exposure to it commonly occurs in animal husbandry. Previous studies have shown that LPS infection disturbs steroidogenesis, including progesterone production, and subsequently decreases animal reproductive performance. However, little information about the underlying mechanisms is available thus far. In the present study, an *in vitro*-luteinized porcine granulosa cell model was used to study the underlying molecular mechanisms of LPS treatment. We found that LPS significantly inhibits progesterone production and downregulates the expressions of progesterone synthesis-associated genes (StAR, CYP11A1, and 3*β*-HSD). Furthermore, the levels of ROS were significantly increased in an LPS dose-dependent manner. Moreover, transcriptional factors GATA4 and GATA6, but not NR5A1, were significantly downregulated. Elimination of LPS-stimulated ROS by melatonin or vitamin C could restore the expressions of GATA4, GATA6, and StAR. In parallel, StAR expression was also inhibited by the knockdown of GATA4 and GATA6. Based on these data, we conclude that LPS impairs StAR expression via the ROS-induced downregulation of GATA4 and GATA6. Collectively, these findings provide new insights into the understanding of reproductive losses in animals suffering from bacterial infection and LPS exposure.

## 1. Introduction

Following the preovulatory luteinizing hormone (LH) surge, follicular granulosa cells (GCs) are converted to large luteal cells and promote corpus luteum (CL) formation [1]. Simultaneously, there are changes in steroidogenesis such as the rapid suppression of estrogen synthesis-associated gene CYP19A1 and the elevated expression of the steroidogenic acute regulatory protein (StAR), which is a rate-limiting enzyme in progesterone synthesis [2]. With the help of StAR, serum cholesterol is transported into the mitochondrial inner membrane, where it is transformed into pregnenolone by P450scc (encoded by the CYP11A1 gene) and finally catalyzed into progesterone by 3*β*-HSD [3]. This functional shift-induced high progesterone production plays a crucial role in follicle rupture and the subsequent CL development [4, 5]. Moreover, it also facilitates embryo implantation and leads to successful pregnancies [6]. Otherwise, the inhibition of StAR, CYP11A1, or 3*β*-HSD expression and progesterone production would result in a low conception rate and poor reproductive performance.

During animal management, animals often encounter numerous unfavorable conditions, such as bacterial infection, that may disturb the endocrine system and cause hormonal imbalance. Bacterial infection commonly occurs in animals and disrupts ovarian function [7]. *Escherichia coli* is one of the major bacterial species associated with tissue pathology resulting from the bacterial endotoxin, typically lipopolysaccharide (LPS) [8]. Clinical data showed that LPS has been detected in the follicular fluid or the serum of animals and patients suffering from sepsis and Crohn's disease [9, 10]. LPS concentration in the follicular fluid of healthy animals is about 0.06 ng/mL, whereas, in bacteria-infected animals, it sharply increases to about 176.1 ± 112 ng/mL and in some instances even reaches up to 875.2 ng/mL [11]. Several studies by our group and other researchers have shown that LPS suppresses estradiol production in GCs, decreases the expressions of gonadotropin receptors and CYP19A1 [11–14], and also induces failure of blastocyst implantation [15]. *In vivo* studies have also indicated that LPS could decrease serum progesterone levels and lead to luteolysis of premature CL [16]. Thus, it is now well accepted that bacterial infection-induced LPS contamination in animal husbandry is one of the major factors that hamper reproduction.

Reactive oxygen species (ROS) are a number of reactive molecular byproducts and free radicals that are produced during mitochondrial electron transport in aerobic respiration. In mammalian cells, ROS could be generated in numerous situations, including toxin exposure and bacterial infection [17–19]. The surged ROS cause many deleterious events, including cell death and aging. Recently, several researchers have established that ROS are also involved in cell signaling, including gene expression regulations [20]. In certain situations, ROS serve as beneficial factors, such as in the early onset of infections where the release of ROS is considered an essential part of immediate immune reaction [19]. Additionally, in mammalian ovaries, ROS play positive roles in reproductive biology. For example, ROS may induce rupture in the follicle undergoing ovulation. Nevertheless, excessive ROS-induced oxidative damage disturbs oocyte development and steroidogenesis in granulosa cells, and it is even implicated in premature luteolysis [21, 22]. This evidence indicates that ROS negatively regulate steroidogenesis.

Steroidogenic factor-1 (SF-1) encoded by the nuclear receptor 5A1 (NR5A1) gene is at the helm of the steroidogenic expression program in endocrine organs [23]. Similarly, GATA factors act as key regulators in several tissues and organs. Presently, six GATA transcription factors have been identified in vertebrates, and two of them, GATA4 and GATA6, are found in Sertoli and Leydig cells in the testis [24]. Moreover, GATA4 and GATA6 participate in the testis development and steroid biosynthesis [25, 26]. Previous studies have demonstrated that these three transcriptional factors (SF-1, GATA4, and GATA6) also play critical roles in the development and function of the ovary [27–30], and their knockdown could inhibit StAR expression and progesterone production [31, 32].

Taken together, the aforementioned findings revealed that LPS-induced ROS could impair progesterone biosynthesis of CL and disturb pregnancy maintenance. However, the underlying regulatory mechanism and the roles of NR5A1, GATA4, and GATA6 played in ROS-downregulated progesterone biosynthesis are largely unknown. Therefore, in the present study, *in vitro*-luteinized porcine granulosa-lutein cells (pGL) were used as a model system and were treated with LPS, as well as the ROS scavengers melatonin and vitamin C (Vc). Subsequently, the effects of LPS on progesterone biosynthesis and the expressions of transcriptional factor genes (NR5A1, GATA4, and GATA6) and steroidogenic genes (StAR, CYP11A1, and 3*β*-HSD) were investigated.

## 2. Materials and Methods

### 2.1. Granulosa Cell Isolation, Culture, and *In Vitro*-Induced Luteinization

Ovaries of prepubertal gilts aged 170~180 days were obtained from a local slaughterhouse and transported to the laboratory in a vacuum thermos flask in sterile physiological saline at 37°C within 2 h of isolation. After the ovaries were washed three times with sterile physiological saline at 37°C, follicular fluid and GCs were aspirated from medium-sized follicles containing clear follicle fluid, by using a 10 mL syringe with a 20-gauge needle. The follicular fluid and GC mixture was then transferred to a 15 mL centrifuge tube and then centrifuged at 800 g for 3 min, and the supernatant was discarded. The cells were resuspended, and 1 mL of 0.25% trypsin with EDTA was added to digest cell clumps. Following incubation at 37°C for 3~5 min to disperse clumps of cells, 1 mL of 10% fetal bovine serum- (FBS-) supplemented Dulbecco's modified Eagle's medium/Ham's F-12 nutrient mixture (DMEM/F12, without phenol red) was added to the tube to terminate trypsin digestion. The cells were then centrifuged at 800 g for 15 min to be precipitated and then washed twice with phosphate-buffered saline (PBS). Cell density was adjusted to 2 × 10^6^ cells per well in a 6-well plate in 2 mL of culture medium containing 10% FBS and incubated under a humidified atmosphere containing 5% CO_2_ at 37°C. 24 h after, the cells were then washed with PBS to remove any unattached cells. For the *in vitro* luteinization, cells were treated with 100 IU/mL human chorionic gonadotropin (hCG) as reported elsewhere [33].

### 2.2. LPS and H_2_O_2_ Challenge

For LPS and H_2_O_2_ treatment, after removing unattached cells, the cell culture medium was replaced with fresh DMEM/F12 medium containing 2% FBS and 100 IU/mL hCG and supplemented with different final concentrations (0 ng/mL, 500 ng/mL, 1000 ng/mL, or 2000 ng/mL) [34] of LPS (Sigma: *E. coli* serotype 055:B5) and 0.2 mM or 0.4 mM H_2_O_2_ as described elsewhere [35, 36], respectively. After 24 h incubation in a humidified atmosphere containing 5% CO_2_ at 37°C, cells and culture medium were harvested for further analyses. Cells incubated in the medium without LPS or H_2_O_2_ were considered negative controls.

### 2.3. Cell Viability Assay

pGL were cultured in 96-well plates, and their viability was assessed by utilizing the CCK-8 cell viability assay kit (Cell Counting Kit-8; Shanghai Qcbio Science & Technologies Co. Ltd., Shanghai) according to the manufacturer's instructions after heat treatment; the optical density of the yellow color was measured at 490 nm by using a BioTek Eon microtiter plate reader. The cell viability was expressed as the proportion of absorbance values compared to the control. Three separate experiments were performed on different cultures, and each sample was assayed in triplicate.

### 2.4. RNA Extraction, Reverse Transcription (RT), and Quantitative Polymerase Chain Reaction (qPCR)

Total RNA was isolated from cultured pGL using the RNeasy Mini Kit (Qiagen). One microgram of total RNA from each sample was transcribed into cDNA using the SuperScript III First-Strand Synthesis System (Invitrogen) according to the manufacturer's instructions. Real-time quantitative polymerase chain reaction was performed to quantify the mRNA expression levels of *β*-actin, StAR, CYP11A1, 3*β*-HSD, NR5A1, GATA4, and GATA6 in porcine granulosa-lutein cells (the primer information is shown in Table 1). PCRs were carried out in a 20 *μ*L reaction volume containing SYBR Green I Master Mix (TaKaRa, China). An ABI 7500 system (Applied Biosystems; Foster City, CA, USA) was used to detect the amplification products. Upon completion of the real-time qPCR, threshold cycle (Ct) values were calculated by the ABI 7500 software V.2.0.6 (Applied Biosystems; Foster City, CA, USA). The levels of gene expression were expressed in the comparative Δ method using the formula (1 + *E*)^−ΔΔCt^ and normalized to the expression levels of the *β*-actin internal housekeeping gene (in pilot experiments, the stability of 2 candidate housekeeping genes, *β*-actin and 18S, in different treatments was tested and analyzed using BestKeeper 1, as described by Pfaffl et al. [37]. Results showed that *β*-actin appears to have a good stability, and these data are shown in supplement table 1 and the amplification efficiency of the primers used in this study is shown in supplement table 2). Three separate experiments were performed on different cultures, and each sample was assayed in triplicate.

### 2.5. Measurement of Secreted Progesterone

After treatments, the cell culture medium was assayed immediately or stored at -20°C until assayed. The progesterone levels in the culture medium were measured following the manufacturer's instructions using a competitive enzyme immunoassay kit (Beijing North Institute of Biological Technology, Beijing, China). The inter- and intra-assay coefficients of variation for this assay were less than 15%, and the standard curve ranged from 0.2 to 20 ng/mL. Progesterone levels were normalized to the genome DNA of the corresponding wells as described by Silva et al. [38]. Briefly, after treatment, culture medium was collected for progesterone analysis, and the cells were collected for DNA extraction. The final progesterone levels were analyzed using the following equation: Progesterone levels (ng/mL/ngDNA) = progesterone concentration in culture medium/total amount of DNA. Each sample was measured in triplicate, and 3 separated experiments were performed. Because of the variation of progesterone levels in the separated LPS or H_2_O_2_ treatment experiments, declined progesterone ratios were presented in the final data.

### 2.6. Small Interfering RNA Transfection

GATA4 and GATA6 transient knockdown assays were performed with specific siRNA (siGATA4—F: 5′-CCCAAGAACCUUAACAAAUTT-3′ and R: 5′-AUUUGUUAAGGUUCUUGGGTT-3′—and siGATA6—F: 5′-GCUCUGGUAAUAGCAAUAATT-3′ and R: 5′-UUAUUGCUAUUACCAGAGCTT-3′), and the control group was transfected with nontargeting control siRNA (siControl). The pGL were precultured to 50% confluence in antibiotic-free DMEM/F12 medium containing 10% FBS and then were transfected with 25 nM siRNA using Lipofectamine RNAiMAX in Opti-MEM (Life Technologies) according to the manufacturer's instructions. After 24 h transfection, the expressions of transcriptional factor genes (GATA4, GATA6, and NR5A1) and steroidogenic genes (StAR, CYP11A1, and 3*β*-HSD) were analyzed by using qPCR.

### 2.7. ROS Elimination

To investigate the effect of ROS on the expressions of the steroidogenic genes and the transcriptional factors, LPS- or H_2_O_2_-induced ROS were eliminated by using two different ROS scavengers, melatonin and Vc, respectively. Briefly, *in vitro*-induced pGL were incubated in DMEM/F12 supplemented with 10 mM melatonin [39] or 5 mM Vc [40] in the presence of LPS (1000 ng/mL) or H_2_O_2_ (0.4 mM), respectively.

### 2.8. ROS Detection and Analysis

The intracellular ROS levels in the cells after LPS or H_2_O_2_ treatment were detected with cell-permeant 2′,7′-dichlorodihydrofluorescein diacetate (H2DCFDA; Beyotime Institute of Biotechnology, China) as described elsewhere [41]. Briefly, granulosa cells were seeded on the coverslips in 24-well plates (sterilized coverslips were placed in the well before seeding) and treated as mentioned before and then incubated in H2DCFDA/PBS solutions (1 : 1000) at 37°C for 30 min. After thorough washes in DPBS, coverslips were mounted on glass slides (with cell side laid face down to the glass slide). Finally, the cells were assayed immediately with a confocal microscope (Zeiss LSM700 META). For the ROS level analysis, the mean pixel intensity of 3 different fields of each separated experiment was analyzed (all the cells in each field were analyzed), and the regions next to cells that have no fluoresce were set as the background.

### 2.9. Statistical Analysis

The Prism software (GraphPad Software Inc., San Diego, CA) was used to perform one-way ANOVA followed by Tukey's multiple comparison tests. The results are presented as the mean ± SEM of at least three separate experiments performed on different cultures. Data were considered significantly different from each other if *P* < 0.05.

## 3. Results

### 3.1. LPS Downregulated StAR Expression and Progesterone Production in pGL

**Figure 1:**
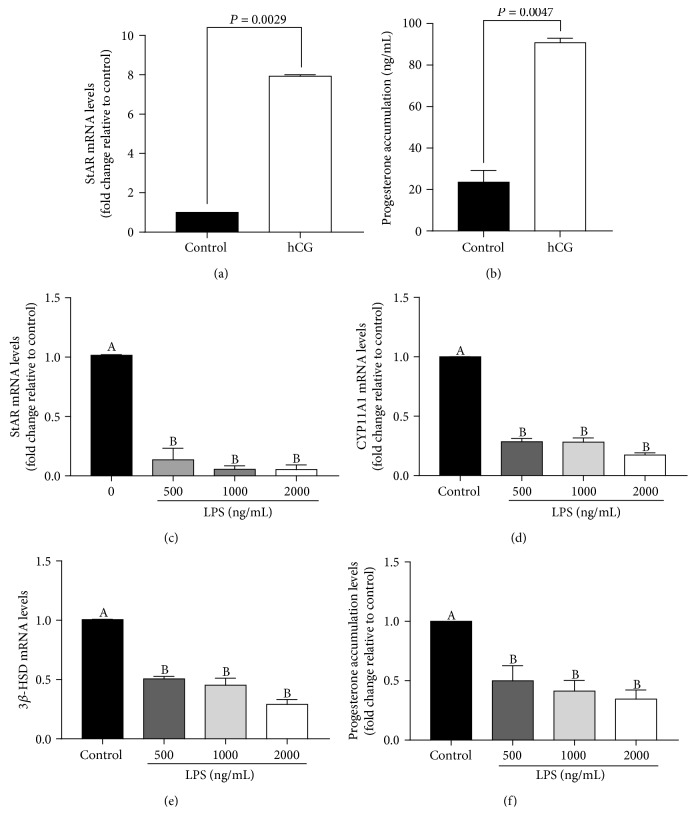
LPS inhibits progesterone synthase expressions and progesterone production in pGL. (a) pGL were treated for 24 h with vehicle control or 100 IU/mL of hCG, and StAR mRNA levels were examined by RT-qPCR. (b) pGL were treated for 24 h with vehicle control or 100 IU/mL of hCG, and progesterone accumulation levels in culture medium were analyzed by ELISA. (c) pGL were treated for 24 h with vehicle control or different concentrations (500, 1000, or 2000 ng/mL) of LPS, and StAR mRNA levels were examined by RT-qPCR. (d) Porcine granulosa-lutein cells were treated for 24 h with vehicle control or different concentrations (500, 1000, or 2000 ng/mL) of LPS, and CYP11A1 mRNA levels were examined by RT-qPCR. (e) pGL were treated for 24 h with vehicle control or different concentrations (500, 1000, or 2000 ng/mL) of LPS, and 3*β*-HSD mRNA levels were examined by RT-qPCR. (f) pGL were treated for 24 h with vehicle control or different concentrations (500, 1000, or 2000 ng/mL) of LPS, and progesterone accumulation levels in culture medium were assayed by ELISA. The results are expressed as the mean ± SEM of at least 3 independent experiments, and values labeled with different letters are significantly different (*P* < 0.05).

First of all, the effectiveness of the *in vitro*-luteinized pGL model was examined by treating it with 100 IU/mL hCG. As shown in Figure 1(a), StAR mRNA levels were sharply upregulated (almost 8 times with respect to control) after a 24-hour hCG treatment. Accordingly, progesterone accumulation in cell culture medium was also significantly increased (Figure 1(b)). These results demonstrated that the *in vitro*-luteinized pGL possess the steroidogenic functions as that of the luteal cell *in vivo*. Thus, these cells offer a good model system for use in future studies.

To check whether LPS plays an inhibitory role in progesterone production, dose-dependent effects of LPS on StAR, CYP11A1, and 3*β*-HSD expressions in pGL were examined. As shown in Figure 1(c), at the concentration of 500 ng/mL LPS, StAR mRNA levels significantly decreased, and the effect was sustained with increasing doses (1000 and 2000 ng/mL). Moreover, mRNA expressions of CYP11A1 and 3*β*-HSD (Figures 1(d) and 1(e)) and progesterone production were all significantly hampered (Figure 1(f)).

### 3.2. LPS Stimulates ROS Generation in pGL

**Figure 2:**
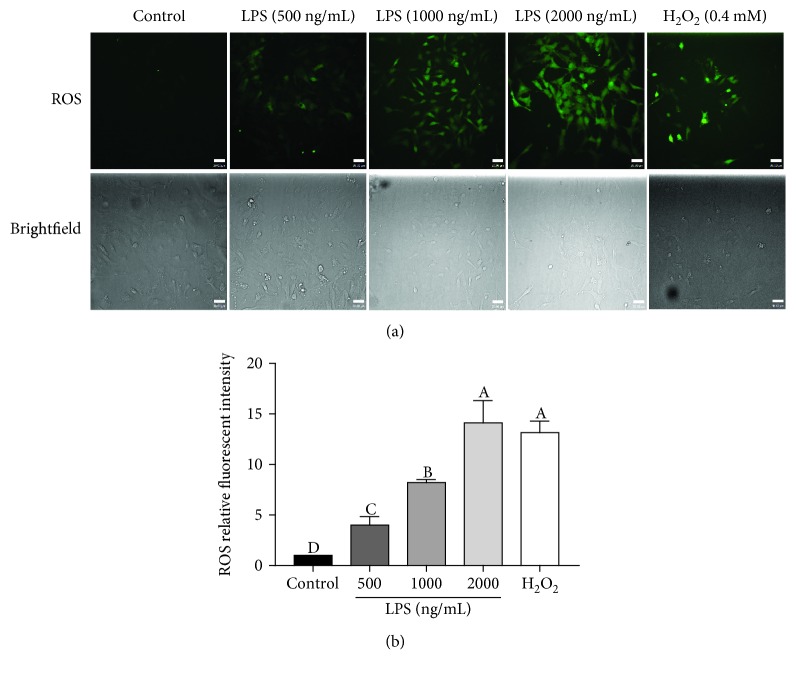
LPS stimulates ROS generation in pGL. (a) pGL were treated for 24 h with vehicle control or different concentrations (500, 1000, or 2000 ng/mL) of LPS and 0.4 mM H_2_O_2_. Intracellular ROS were evaluated by H2DCFDA detection, and fluorescent images are shown (scale bar: 30 *μ*m). (b) After LPS and H_2_O_2_ treatment, intracellular ROS levels were evaluated. The results are expressed as the mean ± SEM of at least 3 independent experiments, and values labeled with different letters are significantly different (*P* < 0.05).

Since it is well established that the generation of inflammatory mediators is central to LPS-induced inflammations, we investigated whether LPS induces ROS generation by using H2DCFDA as a ROS indicator. As shown in Figure 2(a), the intracellular levels of ROS gradually increased in a dose-dependent manner after LPS treatment for 24 hours. Here, cells treated with 0.4 mM H_2_O_2_ were used as a positive control. The mean fluorescent intensity of ROS was also quantified (Figure 2(b)). These results suggested that ROS generation could be induced by the treatment with LPS and H_2_O_2_. Based on these results, we hypothesize that LPS-induced ROS generation is involved in the functional decline of pGL.

### 3.3. H_2_O_2_ Downregulates StAR Expression in pGL

**Figure 3:**
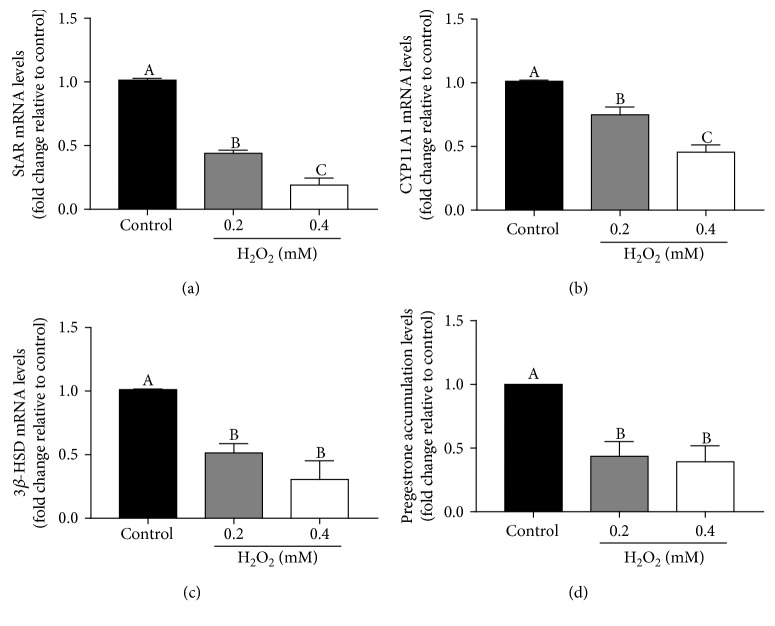
H_2_O_2_ inhibits progesterone synthase expressions and progesterone production in pGL. (a) pGL were treated for 24 h with vehicle control or different concentrations (0.2 or 0.4 mM) of H_2_O_2_, and StAR mRNA levels were examined by RT-qPCR. (b) pGL were treated for 24 h with vehicle control or different concentrations (0.2 or 0.4 mM) of H_2_O_2_, and CYP11A1 mRNA levels were examined by RT-qPCR. (c) pGL were treated for 24 h with vehicle control or different concentrations (0.2 or 0.4 mM) of H_2_O_2_, and 3*β*-HSD mRNA levels were examined by RT-qPCR. (d) pGL were treated for 24 h with vehicle control or different concentrations (0.2 or 0.4 mM) of H_2_O_2_, and progesterone accumulation levels in culture medium were assayed by ELISA. These results are expressed as the mean ± SEM of at least 3 independent experiments, and values labeled with different letters are significantly different (*P* < 0.05).

To test the above hypothesis regarding ROS, the functional changes in pGL were investigated by treatment with 0.2 mM or 0.4 mM H_2_O_2_ in the presence of 100 IU hCG. Results showed that at the concentration of 0.2 mM H_2_O_2_, the levels of StAR mRNA were significantly decreased, and the effect intensified at 0.4 mM H_2_O_2_ (Figure 3(a)). Similarly, the expressions of CYP11A1 and 3*β*-HSD and progesterone production were also significantly downregulated (Figures 3(b)–3(d)).

### 3.4. Transcription Factors GATA4 and GATA6 Are Downregulated after Treatment with LPS and H_2_O_2_

**Figure 4:**
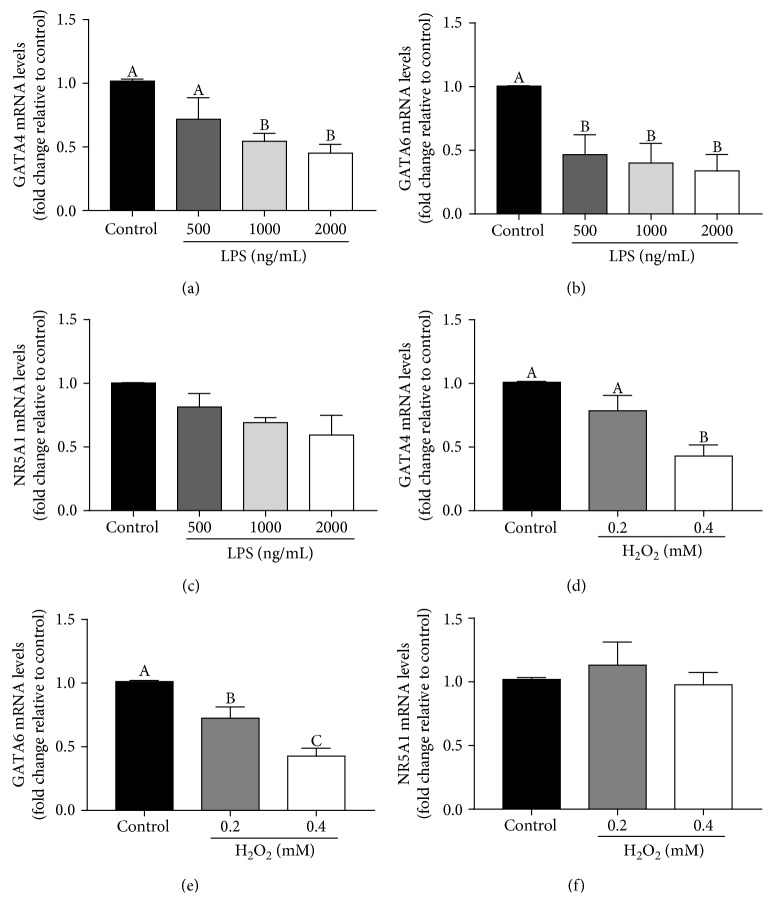
LPS and H_2_O_2_ downregulate the expressions of GATA4 and GATA6, but not NR5A1. (a, b, c) pGL were treated for 24 h with vehicle control or different concentrations (500, 1000, or 2000 ng/mL) of LPS, and GATA4, GATA6, and NR5A1 mRNA levels were examined by RT-qPCR. (d, e, f) pGL were treated for 24 h with vehicle control or different concentrations (0.2 or 0.4 mM) of H_2_O_2_, and GATA4, GATA6, and NR5A1 mRNA levels were examined by RT-qPCR. These results are expressed as the mean ± SEM of at least 3 independent experiments, and values labeled with different letters are significantly different (*P* < 0.05).

The expressions of StAR, CYP11A1, and 3*β*-HSD have been reported to be regulated by the transcription factors GATA4, GATA6, and NR5A1 (SF1) binding to their promoter regions [42–45]. Thus, we further examined the expression profiles of GATA4, GATA6, and NR5A1 when treated with LPS and H_2_O_2_, respectively. The results in Figure 4 show that the expression levels of GATA4 (Figure 4(a)) and GATA6 (Figure 4(b)) significantly decreased after treatment with increasing concentrations of LPS (500 ng/mL, 1000 ng/mL, and 2000 ng/mL) for 24 hours. However, the expression of NR5A1 was not affected (Figure 4(c)). Moreover, we saw similar expression profiles in the H_2_O_2_ treatment (Figures 4(d)–4(f)). These results clearly demonstrated that LPS- and H_2_O_2_-induced ROS may be involved in the downregulation of GATA4 and GATA6, but not NR5A1. Additionally, the expressions of the LPS receptor TLR4 and cell viability after LPS or H_2_O_2_ treatment were also analyzed. Results showed that the expressions of TLR4 were upregulated in the treatment with LPS, but not H_2_O_2_ (as shown in supplement figure 1). Cell viability was increased by the treatment with LPS in a dose-dependent manner. However, it showed opposite results in H_2_O_2_ treatment (as shown in supplement figure 2).

### 3.5. GATA4 and GATA6 Are Necessary in StAR Expression

**Figure 5:**
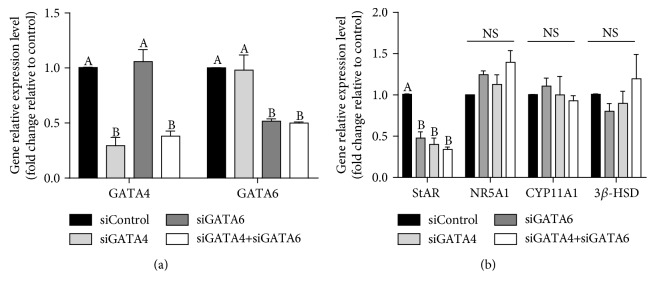
GATA4 and GATA6 are necessary in StAR expression. (a) pGL were transfected for 24 h with 25 nM nontargeting control siRNA (siControl) or 25 nM siRNA targeting GATA4 (siGATA4) and GATA6 (siGATA6). GATA4 and GATA6 mRNA levels were examined by RT-qPCR. (b) mRNA levels of StAR, NR5A1, CYP11A1, and 3*β*-HSD were examined by RT-qPCR after GATA4 and GATA6 knockdown. These results are expressed as the mean ± SEM of at least 3 independent experiments, and values labeled with different letters are significantly different (*P* < 0.05).

To confirm that GATA4 and GATA6 are involved in StAR expressions, knockdown experiments were performed by using siRNA targeting GATA4 and GATA6 (siGATA4 and siGATA6, respectively). First, GATA4 and GATA6 knockdown efficiency was tested. As shown in Figure 5(a), the mRNA levels of GATA4 and GATA6 were significantly decreased by the transfection of siGATA4 and siGATA6, respectively. Moreover, coknockdown of GATA4 and GATA6 showed efficiency comparable to that of single knockdown. Subsequently, the expressions of steroidogenic genes StAR, CYP11A1, and 3*β*-HSD, as well as NR5A1, were tested. As shown in Figure 5(b), the expressions of StAR were significantly downregulated by GATA4 and GATA6 knockdown alone, without LPS or H_2_O_2_ treatment and coknockdown. However, CYP11A1, 3*β*-HSD, and NR5A1 were not affected. These results demonstrated that GATA4 and GATA6, but not CYP11A1 and 3*β*-HSD, are necessary in StAR expression.

### 3.6. Melatonin and Vc Act as ROS Scavengers in pGL

**Figure 6:**
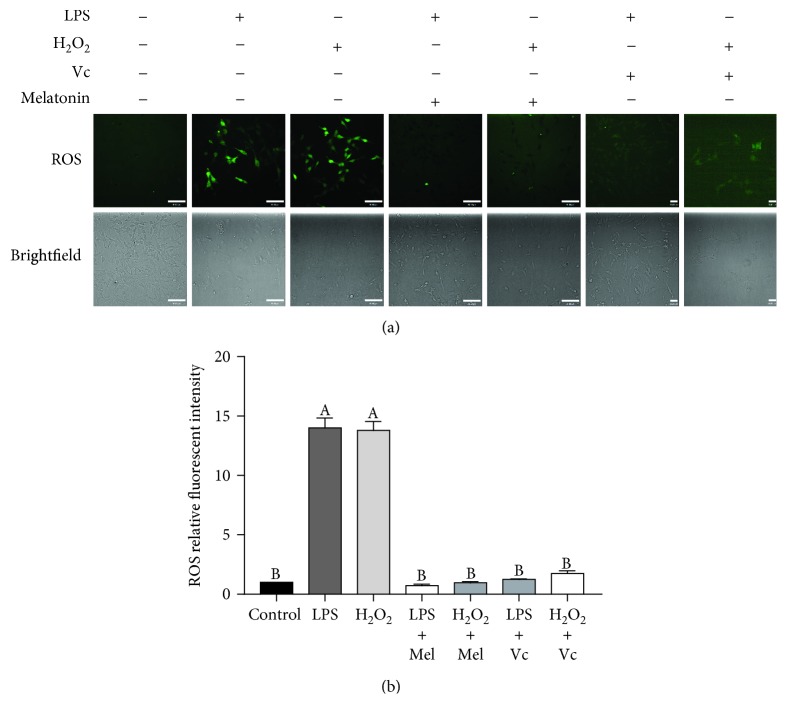
Melatonin and Vc act as ROS scavengers in pGL. (a) pGL were pretreated for 24 h with vehicle control, LPS (1000 ng/mL), or H_2_O_2_ (0.4 mM), with or without melatonin (10 mM) or Vc (5 mM), respectively. Intracellular ROS were evaluated by H2DCFDA detection, and fluorescent images are shown (scale bar: 30 *μ*m). (b) Intracellular ROS fluorescence intensity was evaluated. The results are expressed as the mean ± SEM of at least 3 independent experiments, and values labeled with different letters are significantly different (*P* < 0.05).

As established above, LPS and H_2_O_2_ induce ROS generations. To test whether melatonin and Vc could scavenge the ROS induced by LPS or H_2_O_2_, the cells were treated with 10 mM melatonin or 5 mM Vc in the presence of LPS (1000 ng/mL) or H_2_O_2_ (0.4 mM) for 24 hours. The results of ROS detection showed that ROS levels were significantly eliminated after the supplementation of melatonin or Vc (Figure 6(a)). The mean fluorescent intensity of ROS was also quantified (Figure 6(b)). These findings thus suggested that melatonin and Vc could eliminate intracellular LPS- and H_2_O_2_-induced ROS in pGL.

### 3.7. Elimination of ROS with Melatonin Could Restore the Expressions of StAR, GATA4, and GATA6 in the Presence of LPS or H_2_O_2_

**Figure 7:**
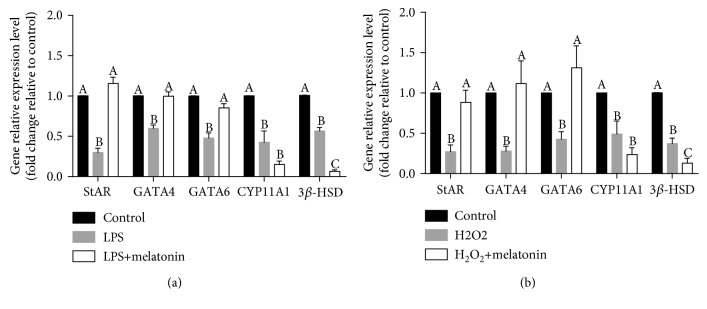
Elimination of ROS with melatonin rescues the expressions of GATA4, GATA6, and StAR after treatment with LPS or H_2_O_2_. (a) pGL were treated for 24 h with vehicle control, LPS (1000 ng/mL), and LPS (1000 ng/mL) with melatonin (10 mM). StAR, GATA4, GATA6, CYP11A1, and 3*β*-HSD mRNA levels were examined by RT-qPCR. (b) pGL were treated for 24 h with vehicle control, H_2_O_2_ (0.4 mM), and H_2_O_2_ (0.4 mM) with melatonin (10 mM). StAR, GATA4, GATA6, CYP11A1, and 3*β*-HSD mRNA levels were examined by RT-qPCR. The results are expressed as the mean ± SEM of at least 3 independent experiments, and values labeled with different letters are significantly different (*P* < 0.05).

To evaluate the effects of melatonin on the expressions of genes used in this study, cells were treated with melatonin alone. Results showed that melatonin alone does not affect the expressions of StAR, NR5A1, GATA4, and GATA6. However, the expressions of CYP11A1 and 3*β*-HSD were downregulated by melatonin. Results are shown in supplement figure 3. To further explore the effect of ROS on StAR, CYP11A1, and 3*β*-HSD expressions, the related transcriptional factors GATA4 and GATA6 were analyzed. The cells were incubated with 10 mM melatonin in the presence of LPS or H_2_O_2_ for 24 hours. As shown in Figure 7(a), StAR, GATA4, and GATA6 expressions were totally restored by the supplementation of melatonin in the presence of LPS. However, CYP11A1 and 3*β*-HSD expressions were not restored. Moreover, we got similar results in the treatment with H_2_O_2_ (Figure 7(b)). These results indicated that LPS decreases StAR expression in pGL, most likely via a ROS-mediated downregulation of the GATA4 and GATA6 signaling pathway.

### 3.8. Vc Treatment Could Also Restore the Expressions of StAR, GATA4, and GATA6 in the Presence of LPS or H_2_O_2_

**Figure 8:**
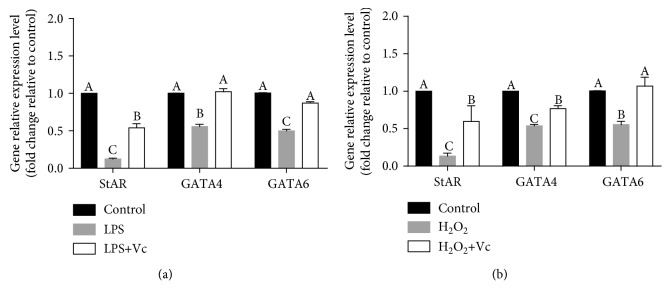
Elimination of ROS with Vc rescues the expressions of GATA4, GATA6, and StAR after treatment with LPS or H_2_O_2_. (a) pGL were treated for 24 h with vehicle control, LPS (1000 ng/mL), and LPS (1000 ng/mL) with Vc (5 mM). StAR, GATA4, and GATA6 mRNA levels were examined by RT-qPCR. (b) pGL were treated for 24 h with vehicle control, H_2_O_2_ (0.4 mM), and H_2_O_2_ (0.4 mM) with Vc (5 mM). StAR, GATA4, and GATA6 mRNA levels were examined by RT-qPCR. The results are expressed as the mean ± SEM of at least 3 independent experiments, and values labeled with different letters are significantly different (*P* < 0.05).

To further confirm the above conclusion that ROS inhibits StAR expression via downregulation of GATA4 and GATA6, the other antioxidant, Vc, was tested in the presence of LPS or H_2_O_2_. First, the effects of Vc on the expressions of genes used in this study were examined by treating cells with Vc alone. Results showed that Vc alone does not affect any of the genes used in this study. Results are shown in supplement figure 3. We further investigated the expressions of StAR, GATA4, and GATA6 with Vc treatment in the presence of LPS or H_2_O_2_. The results indicated that LPS- or H_2_O_2_-induced downregulation of StAR, GATA4, and GATA6 was abolished by Vc treatment (Figure 8). These results reconfirmed the conclusion that ROS-induced downregulation of GATA4 and GATA6 plays a key role in StAR expression declines in the treatment with LPS.

## 4. Discussion

In this study, we used *in vitro*-luteinized porcine granulosa-lutein cells as the model and observed the effects of treatment with LPS on progesterone production, progesterone synthase gene expressions, and their regulatory transcription factors, as well as the possible mechanisms. We found that treatment with LPS induced ROS production in pGL, significantly reduced progesterone production, and significantly reduced gene expression levels of progesterone synthases, such as StAR, CYP11A1, and 3*β*-HSD. We further demonstrated that StAR declines after LPS treatment, which was associated with the downregulation of GATA4 and GATA6, but not NR5A1. These LPS-induced inhibitory effects can be reproduced when treated with H_2_O_2_. Reduction of LPS- or H_2_O_2_-induced ROS by treatment with melatonin restored gene expressions of GATA4, GATA6, and StAR, but not CYP11A1 and 3*β*-HSD. Furthermore, another antioxidant, Vc, showed similar effects on ROS inhibition of StAR, GATA4, and GATA6 expressions. These results indicated that LPS reduces progesterone production through ROS, which, in turn, inhibits StAR through downregulations of GATA4 and GATA6.

Progesterone is essential for early embryo survival and implantation [46]. Thus, to promote these physiological changes, luteinization leads to an important change, which is replacement of estrogen synthesis in the granulosa cells by progesterone produced in the incipient CL cells [47, 48]. The functional changes and cellular remodeling that characterize luteinization are the result of differential expression of genes. As noted earlier, key genes for steroidogenic proteins including StAR, CYP11A1, and 3*β*-HSD were highly expressed in the luteal cells [47, 48]. In addition, transcriptional factors are involved in regulating these genes. Among these, NR5A1, GATA4, and GATA6 were known to be critical for luteinization [31, 49, 50]. Therefore, we supposed that the downregulation of NR5A1, GATA4, and GATA6 may be involved in the inhibition of progesterone production, and this is also a theoretical foundation of the present study.

ROS are often associated with the principle of oxidative stress and induce pathological damage to lipids, proteins, and DNA [51]. In the past 20 years, researchers have concluded that ROS serve as signaling transduction molecules that take part in the regulation of physiological processes [52], including ovarian functions. There are a large number of records of the factors that elevate ROS, such as toxins [53, 54], obesity [55], aging [56], heat stress [57], and endocrine disorder [58]. ROS are involved in the decrease in progesterone production by luteal cells in animals and human beings [21, 59, 60]. Notwithstanding the numerous reproducible results established thus far, the underlying mechanisms, particularly the regulatory role of ROS in the aforementioned transcriptional factors, remain unknown. Studies have shown that LPS, the membrane component from gram-negative bacteria, could stimulate ROS generation in several organs and tissues via TLR4 [61, 62]. Unsurprisingly, some researchers offered a discording result that LPS had no effect on oxidative stress in bovine granulosa cells [63]. To verify whether LPS acts as a ROS stimulus in follicular cells, we treated primary pGL with LPS. Results showed that LPS treatment led to a significant elevation in ROS at a minimum concentration of 500 ng/mL, and the ROS levels were increased with increases in LPS concentrations. With the surge in ROS, steroidogenic genes StAR, CYP11A1, and 3*β*-HSD were inhibited, as previously reported. We also found for the first time that ROS primarily affected the expressions of GATA4 and GATA6, but not NR5A1.

Melatonin is the predominant product of the pineal gland and is distributed in every part of the body including the follicular fluid [64]. It regulates several cell functions, including ovarian functions. Previous data showed that melatonin maintains antral follicular development, stimulates estrogen production in GCs, and promotes follicular maturation [65]. It also acts as an antioxidant directly or via the activation of antioxidative components, such as superoxide dismutase (SOD), by binding to its receptor [66–68]. In the present study, melatonin was used as a scavenger of LPS-induced ROS. After melatonin treatment, StAR expression was totally restored with complete restoration of GATA4 and GATA6. These results would be more convincing if we could show increased expression of CYP11A1 and 3*β*-HSD, as well as the production of progesterone after melatonin treatment. Although tests were done several times, we could not find any significant differences between the control and melatonin-treated cells. Based on these results, the decreased expressions of GATA4 and GATA6 indicate that they may play a role in LPS-induced ROS downregulation of the expression of StAR (but not CYP11A1 and 3*β*-HSD). The conclusion that ROS-induced GATA4 and GATA6 downregulation is predicated in StAR declines was further confirmed by another ROS scavenger, Vc. In view of the fact that CYP11A1 and 3*β*-HSD were not restored, we speculate that other factors or signaling pathways might be involved, but not the ROS/GATA4/GATA6 system. For example, the sirtuin (SIRT) family members are needed in the expressions of CYP11A1 [69], and the expression of Sirt4 can be inhibited by LPS in Leydig cells and cause mitochondrial dysfunction [70]. Whether the expressions of SIRT family members are inhibited by LPS and whether they are involved in the expressions of CYP11A1 and 3*β*-HSD in pGL still need further study. The possible signal transduction in which ROS affect GATA4 and GATA6 expressions is also largely unknown; however, some previous studies have shown some indication of this. In embryonic stem cells, the MAPK pathways (Erk1/2, JNK, and p38) are activated by ROS and inhibit GATA4 expression. Additionally, MAPK activation can be abolished and GATA4 expression was upregulated in the presence of free radical scavengers [71]. Thus, these results gave some clues that there may be some relations between ROS-activated MAPKs and GATA4/GATA6 expression declines.

**Figure 9:**
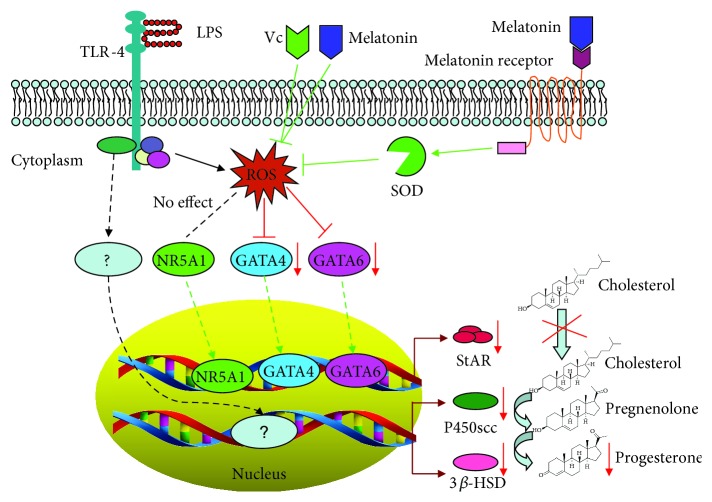
Schematic diagram of the proposed molecular mechanism for LPS-induced downregulation of StAR expression and progesterone production in pGL. Treatment with LPS leads to the elevation of ROS and subsequently reduces GATA4 and GATA6 expressions, which finally reduced the expression of StAR. Elimination of ROS with melatonin or Vc could rescue the expressions of GATA4, GATA6, and StAR. LPS treatment also downregulates P450scc (encoded by the CYP11A1 gene) and 3*β*-HSD expressions, but not through the ROS/GATA4/GATA6 signaling pathway.

In summary, we conclude that bacterial infection or LPS exposure-induced ROS decrease progesterone production in porcine granulosa-lutein cells by inhibiting StAR, CYP11A1, and 3*β*-HSD expressions. During this signaling process, LPS-induced ROS is involved in inhibiting the expressions of GATA4 and GATA6, but not NR5A1, and subsequently in blocking the expression of the progesterone biosynthesis-associated gene StAR, but not CYP11A1 and 3*β*-HSD. The findings of our study provide important and novel insights that contribute to the understanding of signaling pathways that are triggered by LPS and decrease progesterone production in bacterial infection (Figure 9). These results improve and expand upon the basic information on the effects of ROS on ovarian function.

## Figures and Tables

**Table 1:** Primers used in this study.

Gene	Accession no.	Primer sequences (5′-3′)	Annealing	Length
*β*-Actin	XM_003357928.2	F: CTTCCTGGGCATGGAGTCC	60°C	201 bp
		R: GGCGCGATGATCTTGATCTTC		
StAR	AY368628.1	F: CATTACCATCTACTCCCAGC	60°C	109 bp
		R: AACCCGTATCTTTCTTGTCAG		
CYP11A1	NM_214427.1	F:GTCCCATTTACAGGGAGAAGCTCG	60°C	182 bp
		R: GGCTCCTGACTTCTTCAGCAGG		
3*β*-HSD	NM_001004049.1	F: TTCCTGGCAAGTATTTCTCGG	60°C	110 bp
		R: TCCAGCAACAAGTGGACGAT		
NR5A1	NM_214179.1	F: CTGCCTCAAGTTCCTCATTCTC	60°C	122 bp
		R: GGTAGTGGCACAGGGTGTAATC		
GATA4	NM_214293	F: TGAAGCTCCATGGTGTCCC	60°C	150 bp
		R: CTGCTGGAGTTGCTGGAAG		
GATA6	NM_214328	F: AGAAACGCCGAGGGTGAAC	60°C	110 bp
		R: CGTTTCCTGGTCTGAATTCCC		

## Data Availability

The data used to support the findings of this study are included within the article.

## Supplementary Materials

Supplementary Materials Supplement figure 1: LPS, but not H_2_O_2_, treatment upregulates TLR4 expression. Supplement figure 2: cell vitality was increased by LPS but decreased by H_2_O_2_. Supplement figure 3: melatonin and Vc by themselves have no effect on StAR, NR5A1, GATA4, and GATA6 expressions. Supplement table 1: the reference gene stability assay. Supplement table 2: amplification efficiency information of the primers used in this study.
