# Supplementary material for: ROS-Induced GATA4 and GATA6 Downregulation Inhibits StAR Expression in LPS-Treated Porcine Granulosa-Lutein Cells
Source: Oxid Med Cell Longev. 2019 Apr 22;2019:5432792. doi: 10.1155/2019/5432792 (PMC6501234; doi:10.1155/2019/5432792)
Supplement: Supplementary Materials — Supplement figure 1: LPS, but not H2O2, treatment upregulates TLR4 expression. Supplement figure 2: cell vitality was increased by LPS but decreased by H2O2. Supplement figure 3: melatonin and Vc by themselves have no effect on StAR, NR5A1, GATA4, and GATA6 expressions. Supplement table 1: the reference gene stability assay. Supplement table 2: amplification efficiency information of the primers used in this study. [file 5432792.f1.pdf]

ROS induced GATA4 and GATA6 down-regulation inhibits StAR expression in LPS-treated porcine granulosa-lutein cells

Xiaolu Qu[1,2,3], Leyan Yan[1,2], Rihong Guo[1,2], Hui Li[1,2*], Zhendan Shi [1,2*]

1. Institute of Animal Science, Jiangsu Academy of Agricultural Sciences/Key laboratory of Animal Breeding and Reproduction, Nanjing, 210014, China
2. Jiangsu Key Laboratory for Food Quality and Safety-State Key Laboratory Cultivation Base of Ministry of Science and Technology, Jiangsu Academy of Agricultural Sciences, Nanjing, 210014, China.
3. College of Animal Science and technology, Jilin Agricultural University, Changchun, 130118, China

Correspondence should be addressed to Hui Li, lhlydk@hotmail.com

Zhendan Shi, zdshi@jaas.ac.cn

Supplement figure 1. LPS treatment up-regulates TLR4 expression, but not H_2O_2

To clarify the expressions of LPS receptor (TLR4) after LPS or H_2O_2 treatment, pGL were challenged by LPS (1000ng/mL) or H_2O_2 (0.4mM) for 24h. TLR4 expression were assayed by Rt-QPCR using specific primers to pig TLR4 gene (F: 5'-TCAGTTCTCACCTTCCTCCTG-3'; R: 5'-GTTCATTCCTCACCCAGTCTTC-3'). Results showed that TLR4 expression was significantly increased by LPS, but not H_2O_2.

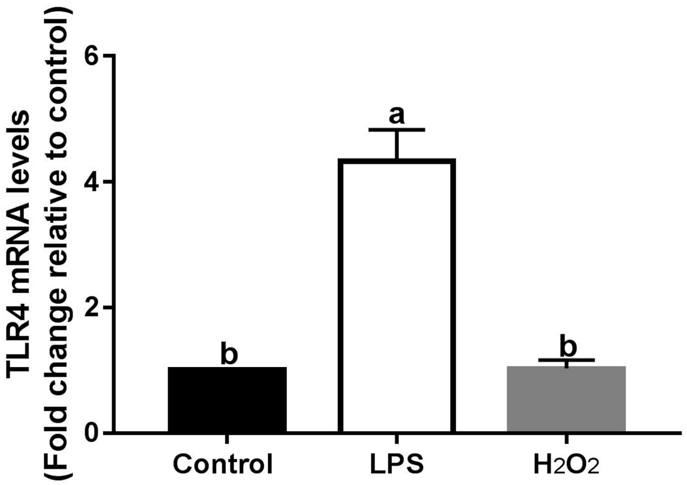

Supplement figure 1. LPS treatment up-regulates TLR4 expression, but not H_2O_2. pGL were treated for 24 h with vehicle control, LPS (1000 ng/mL) or H_2O_2 (0.4mM),TLR4 mRNA levels were examined by RT-qPCR. The results are expressed as the mean ± SEM of at least 3 independent experiments, and values labeled with different letters are significantly different (*P* < 0.05).

Supplement figure 2. Cell vitality was increased by LPS, but decreased by H_2O_2

To clarify the cell vitality in the treatment of LPS or H_2O_2, CCK-8 method was used in the present study as described elsewhere {Li, 2017 #13999}. Results showed that cell viability was increased by the treatment of LPS in a dose-dependent manner. However, it showed an opposite results in H_2O_2 treatment.

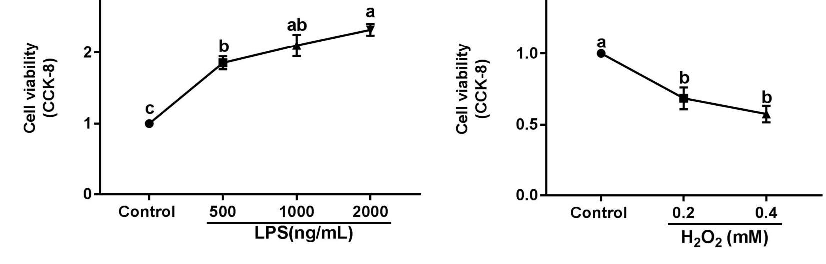

Supplement figure 2. Cell vitality was increased by LPS, but decreased by H_2O_2. pGL were treated

for 24 h with vehicle control, LPS (1000 ng/mL) (A)or H_2O_2 (0.4mM) (B), Cell vitality levels were examined by CCK-8 method. The results are expressed as the mean ± SEM of at least 3 independent experiments, and values labeled with different letters are significantly different ($P < 0.05$).

Supplement figure 3. Melatonin and Vc by themselves have no affect on StAR, NR5A1, GATA4 and GATA6 expressions.

To show that the effects of melatonin and Vc on the expressions of the genes used in this study, pGL were treated with melatonin (10mM) and Vc (5mM) alone for 24h, respectively. After treatment, StAR, NR5A1, GATA4, GATA6, CYP11A1 and 3β-HSD expression levels were analyzed by RT-qPCR. Results shown that Melatonin and Vc by themselves have no affect on StAR, NR5A1, GATA4 and GATA6 expressions, however, the expressions of CYP11A1 and 3β-HSD were down-regulated by melatonin, but not Vc.

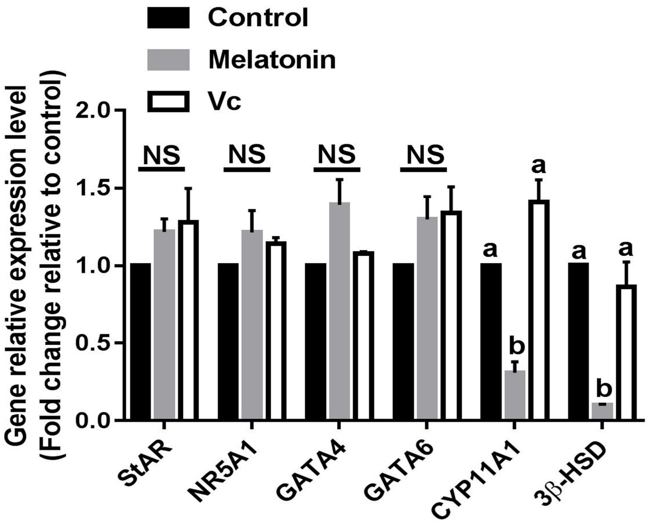

Supplement figure3. pGL were treated by melatonin and Vc for 24h, respectively. The expression levels of StAR, NR5A1, GATA4, GATA6, CYP11A1 and 3β-HSD were examined by RT-qPCR. The results are expressed as the mean ± SEM of 3 independent experiments, and values labeled with different letters are significantly different ($P < 0.05$).

Supplement table 1. The reference gene stability assay.

Actin(CT)	Diff. β-Actin [CP]	Diff. β-Actin[%]	18S(CT)	Diff. 18S [CP]	Diff. 18S[%]
14.85	0.40	2.80	11.76	-0.36	-2.97
14.87	0.42	2.94	12.40	0.28	2.31
14.89	0.44	3.08	11.61	-0.51	-4.21
14.80	0.35	2.45	10.88	-1.24	-10.23
13.77	-0.68	-4.68	13.03	0.91	7.51
13.63	-0.82	-5.65	13.85	1.73	14.28
13.89	-0.56	-3.85	10.51	-1.61	-13.28
13.92	-0.53	-3.64	10.88	-1.24	-10.23
13.98	-0.47	-3.22	14.82	2.70	22.28
15.27	0.82	5.71	10.85	-1.27	-10.48
14.82	0.37	2.59	11.79	-0.33	-2.72
14.20	-0.25	-1.70	10.93	-1.19	-9.82
15.20	0.75	5.22	10.92	-1.20	-9.90
14.40	-0.05	-0.32	15.66	3.54	29.21
14.36	-0.09	-0.59	12.86	0.74	6.11
14.42	-0.03	-0.18	12.51	0.39	3.22

Two house keeping genes (β-Actin and 18S) under same experimental conditions were analyzed by using Bestkeeper 1, as described by Pfaffl [Pfaffl, 2004]. Results shown that β-Actin shows a good stablility.

Supplement table 2.Amplification efficiency information of the primers used in this study

Gene	Slope	Efficiency (E)	R^2
β-Actin	-3.404	96.68%	0.999
StAR	-3.412	96.37%	1
CYP11A1	-3.291	101.3%	0.999
3β-HSD	-3.445	95.1%	0.998
NR5A1	-3.571	90.65%	0.997
GATA4	-5.529	92.02%	1
GATA6	-3.452	94.84%	0.997

Reference

1. Li H, Guo S, Cai L, Ma W, Shi Z. Lipopolysaccharide and heat stress impair the estradiol biosynthesis in granulosa cells via increase of HSP70 and inhibition of smad3 phosphorylation and nuclear translocation. *Cell Signal* 2017,**30**:130-141.
2. Pfaffl MW, Tichopad A, Prgomet C, Neuvians TP. Determination of stable housekeeping genes, differentially regulated target genes and sample integrity: BestKeeper--Excel-based tool using pair-wise correlations. *Biotechnol Lett* 2004,**26**:509-515.
